# Heat shock factor-1 intertwines insulin/IGF-1, TGF-β and cGMP signaling to control development and aging

**DOI:** 10.1186/1471-213X-12-32

**Published:** 2012-11-01

**Authors:** János Barna, Andrea Princz, Mónika Kosztelnik, Balázs Hargitai, Krisztina Takács-Vellai, Tibor Vellai

**Affiliations:** 1 Department of Genetics, Eötvös Loránd University, Pázmány Péter stny. 1/C, Budapest, H-1117, Hungary

**Keywords:** Heat shock factor-1, *C*. *elegans*, Dauer development, Aging, Signaling crosstalk

## Abstract

**Background:**

Temperature affects virtually all cellular processes. A quick increase in temperature challenges the cells to undergo a heat shock response to maintain cellular homeostasis. Heat shock factor-1 (HSF-1) functions as a major player in this response as it activates the transcription of genes coding for molecular chaperones (also called heat shock proteins) that maintain structural integrity of proteins. However, the mechanisms by which HSF-1 adjusts fundamental cellular processes such as growth, proliferation, differentiation and aging to the ambient temperature remain largely unknown.

**Results:**

We demonstrate here that in *Caenorhabditis elegans* HSF-1 represses the expression of *daf*-*7* encoding a TGF-β (transforming growth factor-beta) ligand, to induce young larvae to enter the dauer stage, a developmentally arrested, non-feeding, highly stress-resistant, long-lived larval form triggered by crowding and starvation. Under favorable conditions, HSF-1 is inhibited by crowding pheromone-sensitive guanylate cyclase/cGMP (cyclic guanosine monophosphate) and systemic nutrient-sensing insulin/IGF-1 (insulin-like growth factor-1) signaling; loss of HSF-1 activity allows DAF-7 to promote reproductive growth. Thus, HSF-1 interconnects the insulin/IGF-1, TGF-β and cGMP neuroendocrine systems to control development and longevity in response to diverse environmental stimuli. Furthermore, HSF-1 upregulates another TGF-β pathway-interacting gene, *daf*-*9*/*cytochrome P450*, thereby fine-tuning the decision between normal growth and dauer formation.

**Conclusion:**

Together, these results provide mechanistic insight into how temperature, nutrient availability and population density coordinately influence development, lifespan, behavior and stress response through HSF-1.

## Background

**Figure 1:**
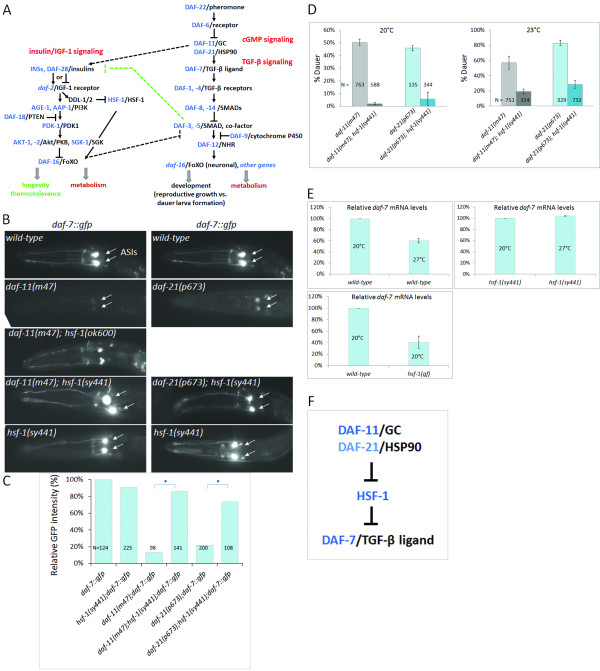
**HSF-1 represses *daf*-*7 *expression, and is negatively regulated by DAF-11 and DAF-21. A**, A signaling network of the *C*. *elegans* insulin/IGF-1, cGMP and TGF-β neuroendocrine systems. The model relies on previously published data. TGF-β signaling promotes reproductive growth. Under adverse environmental conditions, *daf*-*7* becomes downregulated, resulting in the activation of the nuclear hormone receptor DAF-12 that eventually induces dauer development. Crowding pheromone-dependent cGMP signaling mediated by the receptor guanylate cyclase DAF-11 acts upstream of DAF-7 to inhibit dauer larva formation. Insulin/IGF-1 signaling hampers dauer development through the TGF-β pathway. In addition, activity of the IGF-1 receptor DAF-2, which is inhibited or activated by different insulin-like peptides, such as DAF-28, accelerates aging by inhibiting nuclear translocation of the forkhead transcription factor DAF-16. HSF-1 is negatively regulated by insulin/IGF-1 signaling, and required for longevity response triggered by DAF-16. The inhibitory effect of DAF-2/IGF-1 signaling on HSF-1 occurs through the activation of the DDL-1/2 proteins, two negative regulators of HSF-1. DAF-11 upregulates *daf*-*28* (the dotted arrow), while DAF-3 influences DAF-2 activity through its regulation of the *ins*-*7* agonist and *ins*-*18* antagonist (the dotted green bar). Arrows indicate activations, bars represent inhibitory interactions. *C*. *elegans* proteins are in blue; their mammalian counterparts are in black. Dotted lines show known signaling links between these neuroendocrine systems. FoXO: Forkhead box O transcription factor; PDK1: 3-phosphoinositide-dependent kinase 1; Akt: AKT8 virus protooncogene; PKB: protein kinase B; SGK: serum- and glucocorticoid-inducible kinase; PTEN: phosphatase and tensin homolog; PI3K: phosphoinositide 3-kinase; IGF-1: insulin-like growth factor receptor-1; HSF-1: heat shock factor-1; NHR: nuclear hormone receptor; SMAD: *Caenorhabditis elegans* protein small (SMA) and *Drosophila* protein mothers against decapentaplegic (MAD); TGF-β: transforming growth factor-beta; HSP90: heat shock protein 90; GC: guanylate cyclase. **B**, Both DAF-11 and DAF-21 activate *daf*-*7* expression via inhibiting HSF-1. DAF-7 abundantly accumulates in the two ASI neurons (white arrows) in wild-type L1 larvae. In contrast, *daf*-*7* is strongly downregulated in *daf*-*11*(*m47*) and *daf*-*21*(*p673*) mutants (only a faint *daf*-*7* expression is visible in the ASIs). HSF-1 deficiency suppresses *daf*-*7* repression in *daf*-*11*(*m47*) and *daf*-*21*(*p673*) mutant genetic backgrounds. At 20°C, mutations in *hsf*-*1* do not significantly alter *daf*-*7* expression. This implies that HSF-1 has no or a weak activity at this temperature. **C**, Quantification (mean value) of *daf*-*7*::*gfp* expression in the ASIs in wild-type and mutant genetic backgrounds. *indicates p<0.0001. **D**, Dauer development in both *daf*-*11*(*m47*) and *daf*-*21*(*p673*) mutants requires HSF-1 activity. Mutational inactivation of *hsf*-*1* largely protects *daf*-*11*(*m47*) and *daf*-*21*(*p673*) mutant animals from developing as dauer larvae. In each single mutant vs. double mutant comparison, p<0.0001. **E**, Transcriptional activity of *daf*-*7* depends on the ambient temperature and HSF-1 activity. qRT-PCR analysis shows that *daf*-*7* transcript levels decrease at 27°C (the left panel; p<0.001), as compared with those measured at 20°C, and this response requires HSF-1 activity (the right panel; p=0.993). In agreement with these results, hyperactivation of HSF-1 decreases *daf*-*7* mRNA levels at 20°C (bottom; p<0.001). *hsf*-*1*(*gf*) represents a hyperactivating effect of an integrated *hsf*-*1* transgene (*hsf*-*1* cDNA). **F**, Epistasis model showing that DAF-11 and DAF-21 stimulate DAF-7 activity via inhibition of HSF-1. Thus, HSF-1 is an upstream component of the TGF-β cascade; it represses *daf*-*7*, thereby promoting dauer development at high temperatures. In fluorescent figures, images were captured with the same exposure time, and animals were examined at the L1 stage. N indicates number of animals tested, bars represent S.E.M. For statistics: *Student*’*s t*-test.

In the nematode *Caenorhabditis elegans* the insulin/IGF-1 and TGF-β signaling pathways co-regulate metabolism, aging, stress tolerance and development [1-5]. Harsh environmental conditions (e.g., starvation, high population density or high temperatures) and mutations that reduce the activity of either of these pathways cause lipid accumulation in the adipose tissues, lifespan extension, an enhanced tolerance against heat stress, and a switch in the developmental program from normal reproductive growth to dauer development (the dauer is an alternative third larval stage, a developmental diapause, in which the animal shifts metabolism and alters behavior in order to maximize survival and dispersal) [6,7]. The membrane bound receptor guanylate cyclase (GC) DAF-11 (dauer formation constitutive; Daf-c) is an upstream regulator of both pathways. It generates the messenger molecule cGMP, which activates the TGF-β ligand DAF-7 and the insulin/IGF-1 ligand DAF-28 (Figure 1A) [8]. Interestingly, mutations blocking insulin/IGF-1 or TGF-β signaling promote dauer development in a temperature-dependent fashion. For example, at 20°C mutant animals defective for DAF-2 (the worm IGF-1 receptor) or DAF-7 develop as dauer larvae with a relatively moderate penetrance. At 25°C, however, both single mutants develop exclusively as dauer larvae. Thus, expression of the Daf-c phenotype in *daf2*(−) and *daf7*(−) mutants relies on the perception of the ambient temperature. This implies that the effects of nutrient supply, population density and temperature are somehow orchestrated in the animal to influence reproductive growth, longevity, stress resistance and metabolism.

In divergent eukaryotic species, the transcription factor HSF-1 adjusts various cellular processes in response to heat stress through initiating a conserved transcriptional program [9-11]. Upon temperature increase, HSF-1 becomes activated via trimerization and phosphorylation, then translocates into the nucleus to promote the transcription of genes that encode heat shock proteins (HSPs) such as Hsp70, Hsp72, and Hsp90 [12-14]. These factors largely contribute to the protection of cells from protein-damaging stress. In *C*. *elegans*, insulin/IGF-1 signaling inhibits HSF-1 activity [9,15]; DAF-2 was recently shown to inhibit phosphorylation of DDL-1 (DAF-16-dependent longevity) and DDL-2, two negative regulators of HSF-1, in order to keep HSF-1 in an inactive (the DHIC protein complex bound) form [15]. When insulin/IGF-1 signaling is attenuated, HSF-1 and the insulin/IGF-1 signaling target DAF-16/FoXO (Forkhead box-O) stimulate the expression of genes required for longevity and stress resistance (Figure 1A) [1]. Among their common targets, several small heat shock protein encoding genes can be found [1].

## Results

### HSF-1 represses *daf*-*7* expression

Under favorable conditions, DAF-7/TGF-β accumulates in two chemosensory neurons, the ASIs, to promote reproductive growth cell non-autonomously [16]. DAF-11/GC activity, which is inhibited by the crowding (dauer) pheromone DAF-22, leads to *daf7* upregulation (Figure 1A). Indeed, an integrated *daf7*::*gfp* reporter (*ksIs2*) showed a strong ASI-specific expression in an otherwise wild-type, but only a faint expression in a *daf11*(−) mutant genetic background (Figure 1B, C). We found that HSF-1 function is required for *daf7* repression in animals defective for DAF-11: *daf11*(−); *hsf1*(−) double mutants displayed nearly wild-type (i.e., strong) levels of *daf7* expression (Figure 1B, C). A similar result was observed with variable penetrance in *daf11*(−) mutant animals depleted for HSF-1 [on average, 82% of *daf11*(*m47*); *hsf1*(*RNAi*) animals showed wild-type levels of *daf7* expression; N=233]. These results suggest that HSF-1 mediates the regulatory effect of DAF-11 on *daf7* transcription, and that HSF-1 represses *daf7*.

*daf11* shares many functions with *daf21* encoding an Hsp90 chaperone; *daf11* and *daf21* have similar epistasis relationships with other genes in the dauer pathway (Figure 1A) [8,17]. A *daf21* allele, *p673*, causes a massive downregulation of *daf7* expression in the ASIs. This prompted us to monitor whether inactivation of HSF-1 suspends *daf7* repression in *daf21*(*p673*) mutants, too. Expression of *daf7* in the *daf21*(*p673*); *hsf1*(*sy441*) double mutant genetic background was indeed as strong as in the wild-type background (Figure 1B, C).

To confirm the results above, we assayed dauer formation in *daf*-*11*(−) single mutant versus *daf*-*11*(−); *hsf*-*1*(−) double mutant animals, and found that HSF-1 deficiency strongly suppresses dauer development in *daf*-*11*(−) genetic background (Figure 1D). Thus, HSF-1 activity is required for dauer formation in *daf*-*11*(−) mutants animal. Dauer development in *daf*-*21*(*p673*) mutant animals was also blocked by the *hsf*-*1*(*sy441*) mutation [the amorph *hsf*-*1*(*ok600*) mutation arrests development at the L1/L2 larval stages, therefore *hsf*-*1*(*ok600*) mutants were excluded from the dauer formation assays] (Figure 1D). DAF-11/GC and DAF-21/Hsp90 hence upregulate *daf*-*7* through inhibiting its upstream transcriptional repressor, HSF-1. We suggest that HSF-1 is a target of cGMP signaling, and links the cGMP and TGF-β pathways in development control.

Next, we measured *daf*-*7* transcript levels by quantitative RT-PCR. Levels of *daf*-*7* mRNA were reduced by half when animals were grown at 27°C, as compared with those maintained at 20°C (Figure 1D). High temperature-induced downregulation of *daf*-*7* was abolished by the *hsf*-*1*(*sy441*) mutation, suggesting that HSF-1 mediates the modulatory effect of temperature on *daf*-*7* transcription. Moreover, hyperactivation of HSF-1 decreased *daf*-*7* transcriptional activity by half at 20°C (Figure 1E). These data imply that DAF-11 and DAF-21 negatively regulate HSF-1, whose activity in turn represses *daf*-*7* expression (Figure 1F).

**Figure 2:**
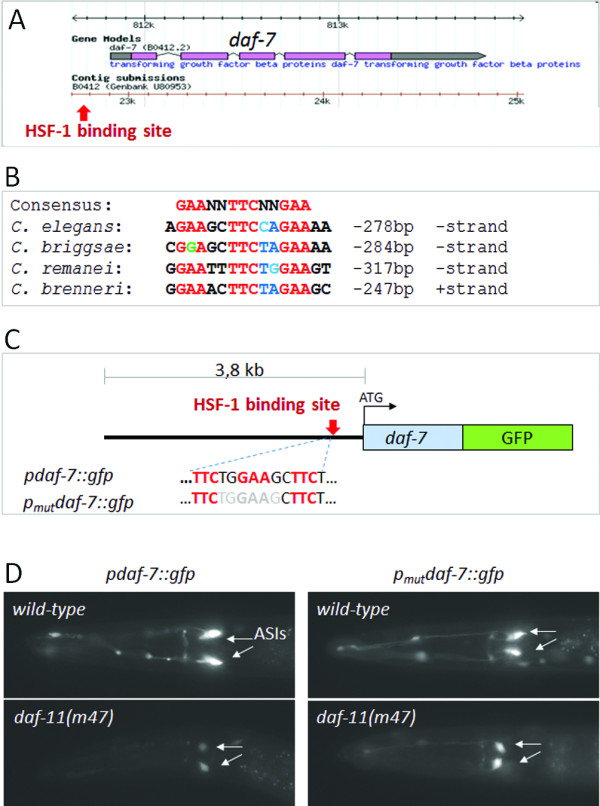
**The upstream regulatory region of *daf*-*7 *contains a conserved HSF-1 binding site that is responsive *in vivo. *A**, The structure of *daf*-*7*; purple boxes represent exonic sequences; grey boxes indicate upstream and downstream regulatory sequences. The position of this conserved HSF-1 binding site is indicated by the red arrow. **B**, This site is highly conserved in the *daf*-*7* genomic environment of *Caenorhabditis* species. Red letters indicate strictly conserved sequences within the consensus HSF-1 binding site. **C**, Scheme of a transcriptional fusion *gfp*-labeled *daf*-*7* reporter (p*daf*-*7*::*gfp*) driven by a 3.8 kb long upstream regulatory sequence. A mutated version of the reporter (p_mut_*daf*-*7*::*gfp*) lacking several nucleotides from the potential HSF-1 binding site (grey letters in the next upper panel) is also shown. **D**, The *daf*-*11*(*m47*) mutation strongly represses the expression of the wild-type reporter (the repression occurs with full penetrance). The promoter-mutated construct, however, is not responsive to the *daf*-*11*(*m47*) mutation: *m47* fails to downregulate p_mut_*daf*-*7*::*gfp*. 82% of these transgenic *daf*-*11*(*m47*) animals showed strong (wild-type levels) *daf*-*7* expression (N=264). This suggests an *in vivo* functionality for this particular HSF-1-binding element.

As HSF-1 acts as a transcription factor, we next asked whether it directly influences *daf7* activity. Our genome-wide sequence analysis identified a canonical HSF-1 binding site (GAANNTTCNNGAA, see Ref. 1) in the regulatory region of *daf7*, 278 base pairs (bp) upstream of the ATG translational initiation site (Figure 2A) (previously, this approach effectively identified substantive DNA-protein interactions; [18-20]). This site is highly conserved in the *daf7* genomic region of closely related *Caenorhabditis* species (Figure 2B). We generated a transcriptional fusion *daf7*::*gfp* (green fluorescent protein) reporter and a mutated version of this reporter in which 6 critical bases were removed from the canonical HSF-1 binding site (p_*mut*_*daf7*::*gfp*) (Figure 2C). Both reporters were strongly expressed in the ASIs (Figure 2D). However, while the wild-type reporter was downregulated in *daf11*(−) single mutants and remained strong in *daf11*(−); *hsf1*(−) double mutants, expression of the mutant reporter was not significantly changed upon DAF-11 deficiency (Figure 2D). These results provide an *in vivo* evidence for the functionality of this particular HSF-1 binding site in the *daf7* promoter. We conclude that HSF-1 acts together with DAF-11, DAF-21 and DAF-7 along the same signaling axis to control reproductive growth. In this cascade DAF-11 and DAF-21 inhibit HSF-1, thereby stimulating *daf7* expression. Under dauer-inducing conditions, HSF-1 becomes active and represses *daf7*. This epistasis model (Figure 1F) may explain why nematodes can develop as dauer larvae at 27°C even when they are well-fed and propagated at a low population density [21].

### HSF-1 also acts downstream of DAF-7/TGF-β to regulate aging and development

**Figure 3:**
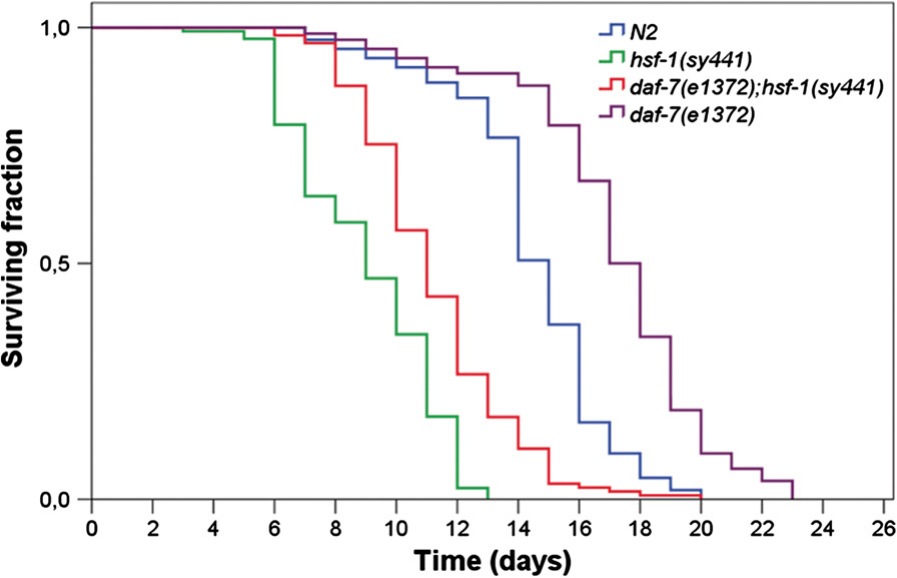
**HSF-1 acts downstream of DAF-7 to regulate lifespan.** HSF-1 is required for the long-lived phenotype of mutant animals defective for TGF-β signaling. HSF-1 deficiency suppresses longevity in *daf*-*7*(*e1372*) mutants. Kaplan-Meyer survival curves were generated by the SPSS software. For comparing *daf*-*7*(−) single mutants vs. *daf*-*7*(−); *hsf*-*1*(−) double mutants or the wild type; p<0.0001 (see the Methods).

TGF-β signaling influences longevity via the insulin/IGF-1 signaling pathway; loss-of-function mutations in *daf7* and *daf4* (*daf4* encodes a type I TGF-β receptor; Figure 1A) can double the animal’s natural lifespan and this longevity effect requires DAF-16 activity [5]. Signaling through DAF-7 negatively regulates the Smad-like transcriptional cofactor DAF-3 (Figure 1A). In the absence of DAF-7 activity, DAF-3 inhibits certain insulin-like peptides and upregulates other insulin-like peptides (indicated by the green broken bar in Figure 1A) to decrease DAF-2/IGF-1 function and extend lifespan [5,22]. As HSF-1 is required for longevity in insulin/IGF-1 signaling deficient animals [1,9], we hypothesized that it mediates lifespan extension in *daf7*(−) mutants as well. Indeed, the long-lived phenotype of *daf7*(*e1372*) mutant animals was strongly, but not completely, suppressed by the *hsf1*(*sy441*) mutation (Figure 3). *daf7*(*e1372*); *hsf1*(*sy441*) double mutants lived nearly as short as *hsf1*(*sy441*) single mutants. This raises the possibility that HSF-1 functions downstream of DAF-7 in ageing control. It is worth to note that the incomplete suppression of longevity in *daf7*(*e1370*) mutants by the *hsf1*(*sy441*) mutation may result from the fact that *sy441* represents a hypomorphic, and not a genetic null, allele.

**Figure 4:**
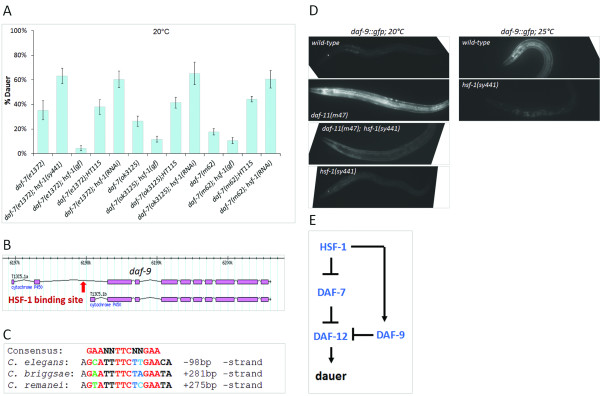
**HSF-1 also acts downstream of DAF-7 to regulate development. A**, Inactivation of HSF-1 enhances, while its hyperactivation decreases, dauer development in *daf*-*7*(−) null mutant genetic backgrounds. Bars represent S.E.M. For each single mutants vs. double mutants or RNAi combination, p<0.001, except for the *daf*-*7*(*m62*) mutant background, where p<0.01 (*Student*’*s t*-test). For each genotype, at least 150 animals were tested. **B**, The structure of *daf*-*9* gene encoding two isoforms. Boxes represent exons, connecting lines indicates introns. A conserved HSF-1 binding site (the red arrow) can be found in the second intron of the longer *daf*-*9* transcript. **C**, This regulatory element is conserved in the *daf*-*9* genomic environment of other *Caenorhabditis* species. Highly conserved nucleotides are in red. **D**, HSF-1 upregulates *daf*-*9*. Fluorescent images showing *daf*-*9*::*gfp* expression in a wild-type, a *daf*-*11*(*m47*) single mutant and a *daf*-*11*(*m47*); *hsf*-*1*(*sy441*) double mutant L2 larva at 20°C. HSF-1 deficiency suppresses hyperactivation of *daf*-*9* in animals defective for DAF-11. Fluorescent images were captured with the same exposure time. 91% of the *daf*-*11*(−); *hsf*-*1*(−) double mutant animals displayed weak (wild-type levels) *daf*-*9* expression (N=166). p<0.0001; *Student*’*s t*-test. Enhanced expression of a *daf*-*9*::*gfp* reporter at 25°C, as compared with that obtained at 20°C. HSF-1 is required for higher temperature-induced ectopic expression of *daf*-*9*. **E**, Our epistasis model showing regulatory interactions among HSF-1, DAF-7 and DAF-9. HSF-1 both promotes (through repressing *daf*-*7*) and inhibits (through upregulating *daf*-*9*) dauer formation. Thus, it acts both upstream and downstream of DAF-7 to modulate development.

Dauer formation in *daf7*(−) null mutant animals correlates with the ambient temperature: the higher the temperature is between 15-25°C, the larger proportion of the population enters the dauer diapause [4]. This phenomenon prompted us to examine whether dysregulation of HSF-1 modulates the ability of these mutants to develop as dauer larvae. We found that inactivation of HSF-1 increases, while its hyperactivation decreases the penetrance of the Daf-c phenotype of DAF-7 deficient animals (Figure 4A). A series of *daf7* mutant alleles, including *e1372*, *ok3125* and *m62*, exhibited an HSF-1-dependent ability to induce dauer development at 20°C. These results imply that HSF-1 acts both upstream and downstream of DAF-7 to modulate the developmental choice between reproductive growth and dauer formation. First, it promotes dauer development by acting downstream of *daf11* and *daf21* to repress *daf7* expression (the upstream effect). Thus, *hsf1* functions between *daf11*/*daf21* and *daf7* in the genetic (TGF-β) pathway that regulates dauer development. Second, HSF-1 inhibits dauer development through interacting with (a) downstream component(s) of the TGF-β pathway (the downstream effect). Interestingly, a previous study also reported that depletion of HSF-1 significantly modulates the percentage of dauer larvae in *daf7*(−) mutant populations [23]. However, the authors showed that RNAi-mediated knockdown of HSF-1 suppresses, rather than enhances (what actually happened in our experiments), the Daf-c phenotype of *daf7*(−) mutant animals. This discrepancy may result from specific RNAi conditions used in that early study. For example, *daf7*(*e1372*) mutants (*e1372* is an amorph mutation) maintained at 25°C could enter the dauer stage with only 20%, although numerous other studies reported that these mutant animals develop as dauer larvae exclusively under identical conditions.

### HSF-1 upregulates *daf*-*9*/*cytochrome P450*

Our data above indicate that HSF-1 may control the expression of a TGF-β component that functions downstream of DAF-7 to affect development. A good candidate for this component is *daf9* that codes for a cytochrome P450 [6,7]. The cytochrome P450 superfamily of enzymes is involved in the metabolism of a large number of organic substances, including synthesis and breakdown of steroid hormones. DAF-9 modulates the activity of the TGF-β dauer formation pathway by inhibiting the nuclear hormone receptor DAF-12 (Figure 1A). Interestingly, *daf9*, which determines two DAF-9 isoforms, is known to be expressed in the hypodermis at early larval stages in a temperature dependent manner (fluorescent images in Figure 4) [6]. Consistent with these results, further sequence analysis identified a conserved HSF-1 binding site in the second intron of *daf9*, which actually is located in the upstream regulatory region of the shorter *daf9* isoform (Figure 4B). This conserved site is also present in the *C*. *briggsae* and *C*. *remanei daf9* loci (Figure 4C). A *daf9*::*gfp* reporter system (*dhEx67*; Ref. 6) containing this potential HSF-1 binding element displayed a faint hypodermal expression in wild-type, but a strong activity in *daf11*(−) mutant background (as DAF-11 deficiency hyperactivates HSF-1) (Figure 4D). Upregulation of *daf9* in *daf11*(−) mutant animals was, however, suppressed by the *hsf1*(*sy441*) mutation, suggesting that HSF-1 mediates temperature-induced stimulation of *daf9*. As DAF-11 accumulates in certain amphid neurons in the head, its negative regulatory effect on the hypodermal *daf9* expression should occur cell non-autonomously. Together, *daf9* may serve as another transcriptional target for HSF-1, and HSF-1 modulates the effect of TGF-β signaling on larval development at multiple points.

### DAF-2/IGF-1 promotes *daf*-*7* expression via inhibition of HSF-1

**Figure 5:**
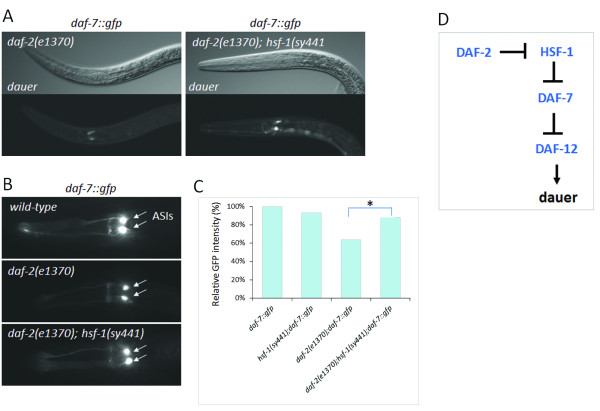
**HSF-1 interconnects insulin/IGF-1 and TGF-β signaling. A**, Inactivation of HSF-1 causes upregulation of *daf*-*7* in *daf*-*2*(*e1370*) mutant dauer larvae (this interaction is fully penetrant; N=462). In the *hsf*-*1*(*sy441*) mutant background, a strong ectopic expression of *daf*-*7* is evident in neurons related to the ventral nerve cord (GFP-positive cells at the ventral side of the body). Up: differential interference contrast images, down: the corresponding fluorescent images. **B**, Activity of the IGF-1 receptor DAF-2 causes *daf*-*7* upregulation via inhibiting HSF-1. The *daf*-*2* mutation *e1370* leads to downregulation of *daf*-*7* at the L1 stage, as compared with the wild-type background. Note that decrease in *daf*-*7* expression in *daf*-*2*(−) mutants is less robust than in *daf*-*11*(−) mutants. The *hsf*-*1*(*sy441*) mutation suppresses downregulation of *daf*-*7* in *daf*-*2*(*e1370*) mutant L1 larvae. 91% of double mutant animals exhibited strong (wild-type levels) *daf*-*7* expression (N=220). In panels **A** and **B**, the corresponding fluorescent images were captured with the same exposure time. **C**, Quantification of *daf*-*7*::*gfp* expression intensity in the ASIs. * indicates: p<0.0001; *Student*’*s t*-test. **D**, Epistatic relationships among DAF-2/IGF-1, HSF-1 and DAF-7/TGF-β. Arrows indicate activations, bars represent inhibitions.

**Figure 6:**
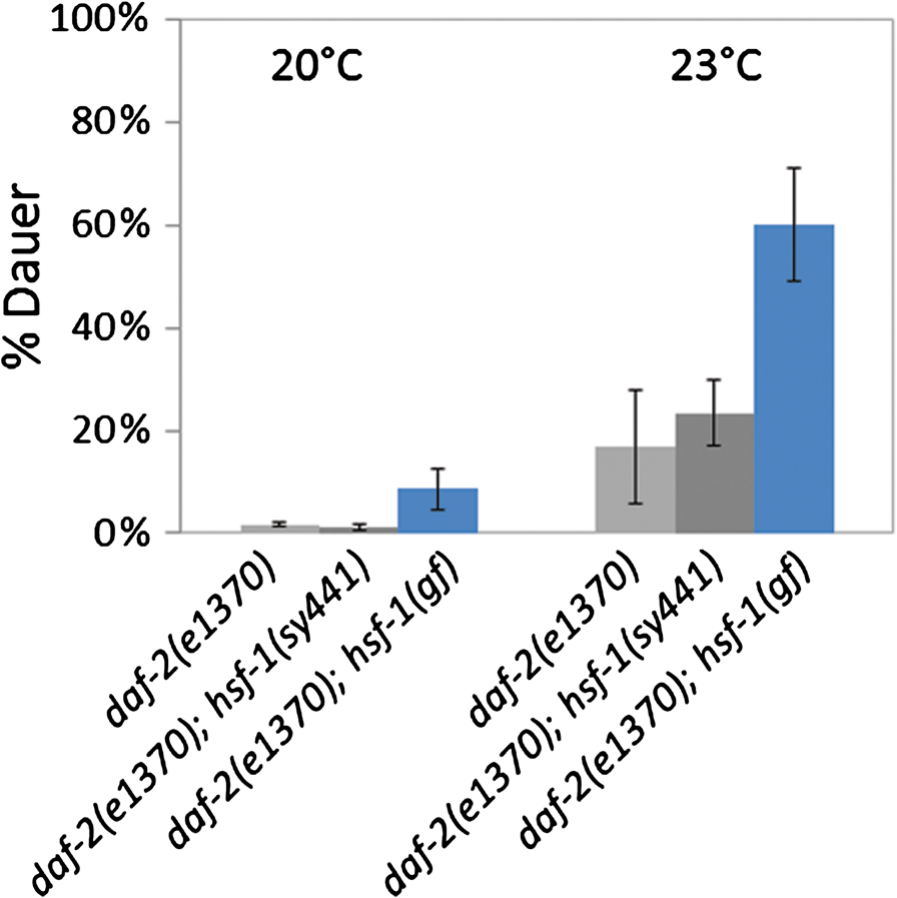
**Hyperactivation of HSF-1 enhances dauer development in *daf*-*2*(*e1370*) mutant background.** This interaction indicates that DAF-2 inhibits HSF-1, which in turn represses *daf*-*7*. Thus, HSF-1 links the insulin/IGF-1 and TGFβ pathways to control the developmental decision between normal reproductive growth vs. dauer larva formation. This epistasis model can explain how starvation induces, while nutrient availability suppresses, dauer development. Bars represent S.E.M. At both temperatures examined, *daf*-*2*(−) vs. *daf*-*2*(−); *hsf*-*1*(*gf*) comparison: p<0.001, *student t*-test.

Insulin/IGF-1 signaling inhibits HSF-1 through modulating the phosphorylation status, and thereby the activity, of the HSF-1 regulator DDL-1/2 proteins [15]. This interaction raises the possibility that DAF-2 also promotes *daf7* expression via HSF-1 (as HSF-1 represses *daf7*). To address this issue we first assayed *daf7* expression in *daf2*(−) mutant dauer larvae (Figure 5A). In these animals, *daf7* was expressed in the ASIs at relatively low levels. In contrast, impairment of HSF-1 function strongly induced *daf7* expression in the ASIs and caused a strong ectopic *daf7* expression in neurons of the ventral nerve cord (Figure 5A). Excessive and ectopic expression of *daf7* was obvious in each *hsf1*(−) mutant dauer larva examined. We next analyzed *daf7* activity in wild-type versus *daf2*(−) mutant background at the L1 stage, and found that *daf7* expression is downregulated upon DAF-2 deficiency (Figure 5B, C). Although a reduction in *gfp* glowing was evident in *daf2*(−) mutant L1 larvae, this expressional change was less robust than in *daf11*(−) mutant L1 larvae. Based on these data one can speculate that DAF-2 only reduces, but does not completely eliminate HSF-1 activity. We also tested the modulatory effect of *hsf1*(*sy441*) mutation on *daf7* expression in *daf2*(−) mutant background. Expression levels of *daf7* in *daf2*(−); *hsf1*(−) double mutants were as nearly strong as in the wild-type background (Figure 3B). This suggests that HSF-1 mediates—via two sequential inhibitory steps—stimulation of *daf7* by DAF-2 (Figure 5B, C). Accordingly, the proportion of dauer larvae in *daf2*(−) mutant populations maintained at 20-23°C was significantly enhanced by HSF-1 hyperactivity (Figure 6). In accordance with previously published data [15], these results point to a regulatory link through which nutritional status of the animal can influence developmental decision between normal reproductive growth and dauer larva formation in a temperature-dependent fashion (Figure 5D).

### HSF-2 promotes reproductive growth

**Figure 7:**
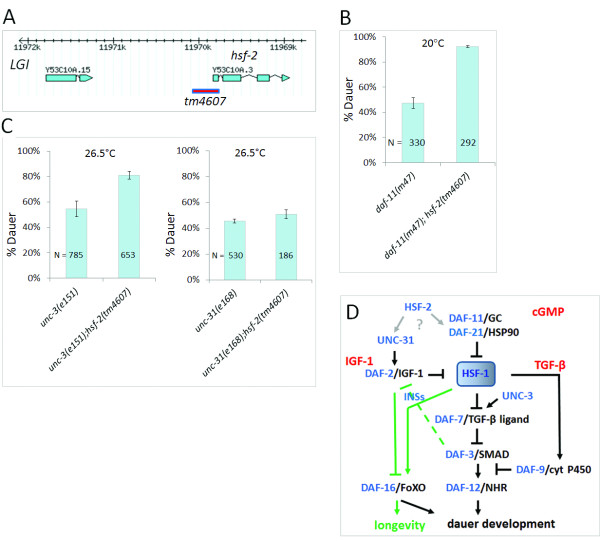
**HSF-2 antagonizes HSF-1 to influence development. A**, The structure of *hsf2*. Blue boxes and interconnecting lines represent exonic and intronic sequences, respectively. The red line represents the extent of *tm4607*, which is a deletion removing upstream regulatory sequences and the first exon of *hsf2*. **B**, *tm4607* promotes dauer development in *daf11*(−) mutants. Thus, HSF-2 acts downstream of, or in parallel to, DAF-11 to influence dauer formation, and antagonizes HSF1 in this function. *hsf2*(*tm6407*) single mutant animals are superficially wild-type; they exhibit neither a dauer formation constitutive phenotype at 25-27°C nor a long-lived phenotype. p<0.0001, *Student*’*s t*-test. **C**, Inactivation of *hsf2* enhances dauer development in *unc3*(−) mutant (p<0.001), but not in *unc31*(−) mutant background (p=0.46), *Student*’*s t*-test. **D**, Our current model showing how HSF-1 interconnects insulin/IGF-1, cGMP and TGF-β signaling to control development and longevity. Arrows indicate activations, bars represents inhibitory regulations. Downstream of HSF-1, regulatory inputs affecting longevity are indicated by green coloring, and regulatory inputs on development are indicated by black arrows and bars. IGF-1: insulin/IGF-1 signaling, TGF-β: TGF-β signaling, cGMP: cGMP signaling. Grey arrows and the question mark indicate that the epistatic position of *hsf2* in this signaling network is uncertain. *hsf2* may act upstream of either *unc31* or *daf11*. Regulatory inputs shown here do not necessarily represent direct interactions. For example, DAF-3 modulates DAF-2 activity (the green dotted bar) through its regulation of the INS-7 (agonist) and INS-18 (antagonist) insulin-like peptides encoding genes [5,22]. In panels **B** and **C**, bars represent S.E.M.

The *C*. *elegans* genome encodes an HSF-1 paralog, HSF-2 (the *WormBase* database; http://www.wormbase.org). *hsf*-*2* (the ORF *Y53C10A*.*3*) is predicted to be composed of 4 exons (Figure 7A). *tm4607* is a deletion allele of *hsf*-*2* which removes nearly 200 bp of upstream regulatory sequences and most part of the first exon. *hsf*-*2*(*tm4607*) mutant hermaphrodites were backcrossed with wild-type males 4 times, and then subjected to a phenotypic characterization at 25 and 27°C. According to our data, the vast majority of *hsf*-*2*(*tm4607*) mutant L1 larvae developed into adulthood at both temperatures, i.e. they did not exhibited a high temperature-induced dauer formation constitutive (Hid) phenotype. Next, we generated an *hsf*-*2*(*tm4607*); *daf*-*11*(*m47*) double mutant strain, and found a significant increase in dauer larva formation in these animals, as compared with *daf*-*11*(*m47*) single mutants (Figure 7B). Thus, unlike HSF-1, HSF-2 inhibits dauer development. Since we do not know whether HSF-2 also functions as a transcription factor, further experiments are needed to clarify the molecular nature of this inhibition.

Inhibitory mutations in *unc3* (uncoordinated) and *unc31* cause a Hid phenotype: the mutant animals do not form dauers at 25°C, but develop as dauer larvae at 27°C [24]. UNC-31 and UNC-3 function upstream of the insulin/IGF-1 and TGF-β signaling pathways, respectively. We assessed whether the *hsf2*(*tm4607*) mutation affects dauer formation in *unc3*(−) and *unc31*(−) mutant animals (Figure 7C). The *tm4607* allele had no influence on dauer development in *unc31*(−) mutants, but significantly increased the ability of *unc3*(−) mutant animals to enter the dauer stage. This synergism suggests that *hsf2* acts in parallel to *unc3*, probably upstream of *unc31*, to promote dauer development (Figure 7D). At the moment, however, we cannot exclude the possibility that *hsf2* may function upstream of *daf11* (indicated by the question mark in Figure 7D). Further epistasis analysis combining *hsf2*(*tm4607*) with *ins*(−), *daf2*(−) and *daf16*(−) mutations would be required to place *hsf2* unambiguously in this signaling network.

## Discussion

Most of the HSF-1 targets identified so far encode heat shock proteins protecting cells from protein-damaging agents [10]. A few examples where HSF-1 transcriptionally represses non-heat shock proteins were also reported [24,25]. In this study we identified two *C*. *elegans* TGF-β pathway-related genes, *daf7*/*TGF*-β and *daf9*/*cytochrome P450*, whose regulatory regions contain a conserved binding site for HSF-1 (Figures 2A, B and 4B, C), and whose expression highly depends on HSF-1 activity (Figures 1B, C, 2D and 4D). *daf7* and *daf9* are likely to be under the direct control of HSF-1. *daf7* expression is repressed, while *daf9* expression is upregulated, by HSF-1, resulting in opposite effects on dauer formation. The overall impact of (dual) HSF-1 activity on this developmental choice (reproductive growth vs. dauer larva formation) is to promote dauer development in the wild type at high temperatures (over 27°C) with only a moderate percentage. Consistent with these results, another cytochrome P450 encoding gene, *cyp35B1*/*dod13*, was recently identified as a HSF-1 target gene [26]. HSF-1 also upregulates *cyp35B1*/*dod13* expression in intestinal cells [26]. Taken together, our present results further suggest that the HSF-1-initiated transcriptional program involves up- and downregulation of “metabolic” genes, the protein products of which have no chaperone function. Rather, these proteins operate as key components of signal transduction pathways implicated in metabolism, development and lifespan control. In the light of these data, one can mechanistically explain how upshift in the ambient temperature affects—in addition to protein homeostasis—diverse cellular processes such as growth, proliferation, fat metabolism, differentiation and aging. Nevertheless, once the developmental decision favoring dauer larva formation is made, several *hsp* genes become also upregulated by HSF-1, and their products contribute to increased stress resistance in the animal.

Our data presented here indicate that HSF-1 represses *daf*-*7* through a direct transcriptional interaction (Figure 2). *daf*-*7* codes for a TGF-β ligand that activates the TGF-β signaling axis promoting reproductive growth. *daf*-*7* regulation by HSF-1 elucidates why nematodes can enter into the dauer stage at high (27°C) temperature even when they are well-fed and not crowded. Moreover, HSF-1 activity is influenced by the receptors DAF-11 and DAF-2 (Figures 1, 5 and 6). DAF-11 is responsive to concentration of the dauer pheromone (i.e., to population density), while DAF-2 senses the animal’s nutritional status. Nematodes deficient in either of these proteins form dauer larvae and age in a temperature-dependent manner. For example, *daf*-*2*(−) mutant animals develop reproductively at 15°C, but develop as dauer larvae at 25°C. More significantly, *daf*-*2*(−) mutants that reach the adulthood live much longer at 15°C than at 25°C. Thus, a complex regulatory interaction mediated by HSF-1 among the TGF-β, guanylate cyclase/cGMP and insulin/IGF-1 signaling systems exists to affect development and lifespan in response to various environmental factors, including population density, nutrient availability and temperature.

HSF-1 stimulates *daf9*/*cytochrome P450* activity (Figure 4B-E), implying that it acts both upstream and downstream of DAF-7 to control nematode development (Figures 4E, 7D). *daf7* repression by HSF-1 promotes, while *daf9* upregulation by HSF-1 suppresses dauer formation under a given condition. Why is a dual function of HSF-1 needed for dauer formation control? At 27°C, only a minor portion (<2-5%) of *C*. *elegans* populations enters the dauer stage [21]. Without stimulating *daf9* activity, HSF-1 would completely inhibit reproductive growth. Actually, this occurs in sensitized [e.g., *daf2*(−) and *daf11*(−)] mutant genetic backgrounds. Since in the temperate and tropical zones the temperature is often above 27°C, nematode populations would frequently exist as non-reproducing dauer larvae under these conditions. This way, HSF-1 regulates both a dauer-inhibiting (*daf7*) and a dauer-inducing (*daf9*) TGF-β pathway component in order to allow the populations to survive (some dauer larvae) and propagate (many reproductive adults) simultaneously under environmental stress, thereby maximizing the chance of the population to survive.

Finally, here we revealed that HSF-1 mediates the regulatory effect of DAF-11 on *C*. *elegans* development. DAF-11 is a receptor guanylate cyclase that is implicated in chemosensation and behavior [8]. Interestingly, several behavioral patterns in various animal species depend on the ambient temperature. Our present finding that guanylate cyclase acts through HSF-1 in controlling *C*. *elegans* dauer formation may help to understand how heat stress, food deprivation or crowding also affects behavior in humans.

## Conclusions

Fundamental insights into how genes and environmental factors influence metazoan metabolism, development and aging have emerged from a genetic dissection of the C. elegans dauer diapause, which is an arrested (non-reproductive), long-lived, and highly stress-resistant larval form triggered by starvation and crowding. Identified molecular pathways that regulate the developmental choice between reproductive growth and dauer larva formation include the insulin/IGF-1, TGF-β and guanylate cyclase/cGMP neuroendocrine systems. Here we show that heat shock factor-1 (HSF-1), a major player in cellular response to heat stress, intertwines these signaling systems in development and aging control. Under adverse environmental conditions HSF-1 promotes dauer larva development through repression of daf-7, which encodes a TGF-β ligand. When conditions are favorable, HSF-1 is inhibited by crowding pheromone-sensitive guanylate cyclase/cGMP and systemic nutrient-sensing insulin/IGF-1 signaling; loss of HSF-1 activity allows DAF-7 to induce reproductive growth. These results provide mechanistic insight into how temperature, nutrient availability and hormonal factors coordinately influence development, stress response, behavior and lifespan through HSF-1. In humans, an orthologous HSF-1-mediated signaling system may be dysregulated in diabetes, cancer and obesity.

## Methods

### Strains and alleles

The wild-type strain corresponds to var. Bristol (N2). The following mutant and transgenic strains were used in this study: PS3551 *hsf*-*1*(*sy441*)*I*, FX04607 *hsf*-*2*(*tm4607*)*I*, CB1370 *daf*-*2*(*e1370*)*III*, CB1372 *daf*-*7*(*e1372*)*III*, RB2302 *daf*-*7*(*ok3125*)*III*, DR62 *daf*-*7*(*m62*)*III*, DP38 *unc*-*119*(*ed3*)*III*, DR47 *daf*-*11*(*m47*)*V*, PR673 *daf*-*21*(*p673*)*V*, CB169 *unc*-*31*(*e169*)*IV*, CB151 *unc*-*3*(*e151*)*X*, FK181 *ksIs2*[*pdaf*-*7*::*gfp* + *rol*-*6*(*su1006*)], VC3071 *hsf*-*1*(*ok600*)/*hIn1*[*unc*-*101*(*sy241*)]I, CF1824 *muEx265*[*hsf*-*1*p::*hsf*-*1cDNA* + *myo*-*3*::GFP], TTV200 *bjIs10*[*hsf*-*1*p::*hsf*-*1cDNA* + *myo*-*3*::GFP] backcrossed 4x, TTV201 *hsf*-*2*(*tm4607*)*I* backcrossed 4x, TTV116 *bjEx1*[p*daf*-*7*::*daf*-*7*::*gfp* + *unc*-*119*(+)], TTV117 bjEx9[p_mut_*daf*-*7*::*daf*-*7*::*gfp* + *unc*-*119*(+)].

### Generation of p*daf*-*7*::*gfp* and p_mut_*daf*-*7*::*gfp* transgenic strains

To generate a p*daf*-*7*::*gfp* reporter, a genomic fragment containing 3,8 kb of the 5’ regulatory region and the first exon of *daf*-*7* was amplified by the following forward and reverse primers: 5’-aaa tcc gag tcc gtg aaa tg-3’ and 5’-aaa cac cgg gag tga aga tg-3’. The resulting fragment was cloned into pGEM®-T Easy vector and subcloned into the vector pPD95.75, using the SphI and SalI restriction enzymes. QuikChange® II Site-Directed Mutagenesis Kit (Stratagene) was used to generate the mutant version of this reporter, with the primers: 5'-caa ttc cgc aaa att ttc tgg ctt ttc tct acg gta tag atg-3' and 5'-cat cta tac cgt aga gaa aag cca gaa aat ttt gcg gaa ttg-3’. These *daf*-*7* genomic fragments were inserted in frame with *gfp* in these constructs. Transgenic strains were generated by biolistic transformation using Biolistic PDS-1000/He particle delivery system (BioRad). 10-15μg linearized plasmid DNA was bombarded onto *unc*-*119*(*ed3*) mutant L4/adult hermaphrodites. Fluorescent images were taken by an Olympus BX51 epifluorescence microscope.

### Quantification of GFP expression intensity

Measurements were performed by the Image J software. Briefly, images were captured with Olympus BX51 epifluorescence microscope with a given exposure time. Cells of interest were selected, and mean grey values were measured. Fluorescent intensity of selected area was calculated by subtracting mean grey value of the background from mean grey value of the object of interest (the same size of areas was selected).

### Lifespan assay

Life span assays were carried out at 25°C, as described previously [27]. Briefly, all strains were maintained at 20°C until the L4 larval stage, then transferred at 25°C, and scored for mean life span. For synchronization, 20–30 gravid well-fed adults were transferred to a new agar plate containing nematode growth medium (NGM) seeded with *E*. *coli* OP50 to lay eggs for 4–5 hours, and then removed. F1 young adults were transferred to NGM plates supplemented with 300μg/μl FUDR (5-fluoro-2'-deoxyuridine). Animals were considered dead when they stopped pharyngeal pumping and responding to touching. SPSS software was used to calculate mean life span and perform statistical analysis. P values for comparing Kaplan-Meyer survival curves between two groups were determined using log-rank (Mantel-Cox) tests.

### Quantification of *daf*-*7* mRNA levels (qRT-PCR)

RNA samples were extracted from 100–200 synchronized L1 larvae, using a PureLink™ Micro-to-Midi Total RNA Purification System (Invitrogene). RNase-free DNase I (Fermentas) was used to eliminate genomic DNA contamination. DNA-free RNA samples were then converted to cDNA, using random hexamers by RevertAid First Strand cDNA Synthesis Kit. Amplification reactions were carried out in a total volume of 20 μl, using LightCycler® FastStart DNA Master SYBR Green I. The reaction conditions for the LightCycler® 2.0 Instrument (Roche) were: 95°C for 5 min, followed by 45 cycles of 95°C for 10s, 60°C for 5s, 72°C for 10s. Fluorescence was measured at each cycle at 72°C. Melting curve analysis was performed after PCR to assess the presence of a unique final product. Gene expression data are presented as the fold change in mRNA transcript abundance in mutant strains, normalized to one endogenous reference gene (*pmp*-*3*), relative to the wild-type strain. Primers for the housekeeping gene *pmp*-*3*: 5’-gtt ccc gtg ttc atc act cat-3’and 5’-aca ccg tcg aga agc tgt aga-3’, and for *daf*-*7*: 5’-caa caa tgt gat agg caa cga-3’and 5’-aac tac gca cgc aca gac ac-3’. The results represent the mean of 3 independent experiments. To compare the means of dauer progeny, variables were analyzed by *Student*’*s t*-test. Results are expressed as mean ± S.E.M.

### Dauer assay

5–10 gravid adults were allowed to lay eggs for 4–6 hours at 20°C to obtain a synchronous population. Plates were placed to the assaying temperatures (20°C, 23°C, 26,5°C), and percentage of dauer larvae was scored typically after 72, 60 and 44 hours, respectively. At least three plates per strain were assayed. Results are expressed as mean ± S.E.M. Data were analyzed by one-way ANOVA and *student t*-test.

### RNA interference

An *hsf*-*1* cDNA clone (*yk1245f10*, a gift of Yuji Kohara) was cut by HindIII and KpnI, and the resulting 1333bp-long cDNA fragment was cloned into the L4440 “feeding” vector. The construct was then transformed into the bacterial strain *Escherichia coli* HT115(DE3).

## Competing interests

The authors declare that they have no competing interests.

## Authors’ contributions

JB, KTV and TV designed research; JB, AP, MK, BH and KTV performed research; JB, KTV and TV analyzed data; JB and TV wrote the paper. All authors read and approved the final manuscript.
